# Our New York Letter

**Published:** 1889-06

**Authors:** M. B. Hutchins

**Affiliations:** New York


### Correspondence.

### OUR NEW YORK LETTER.

### SURGERY.

New York, May, 1889.

The New York *Medical Journal* of May 4th, quotes from an article by Schmid, in "Central blatt für chirugie," describing the success of complete closure of the wound after operation, by the use of iodoform-collodion, and claiming the practicability of good results with no drainage when strict antisepsis is employed. Various operations reported in which the success was good under this method, many of the cases being those in which good drainage is generally thought essential.

The following case is of interest as bearing upon this subject, but as to the cause of failure, it is best to leave your readers to form their own conclusion.

Patient, age 28, carcinoma left breast with sympathetic involvement of axillary lymphatics. Patient thin, of nervous temperament, general health "fair." Incisions elliptical and in direction of fibers of *pectoralis major*. Gland and involved skin removed exposing sheath of *pect. major*. One or two (the affected) axillary glands removed through prolonged incision into axilla. Instrument had been "soaked" in carbolized water 1–40. Small arteries cut were ligated with cat-gut, and oozing of blood checked with towels wrung from carbolized hot water. Edges of incision, in widest part, brought together with three interrupted cat-gut sutures. Beginning at axillary extremity, the edges of incision were sutured with horse hair previously carbolized, the cat-gut being removed as reached. Wound being completely closed and dried, iodoform-collodion was thickly painted over line of suturing. Absorbent cotton and chest bandage applied, left arm being confined to chest. Five hours later, pulse 108; temperature 100. Next morning at 9 a m., pulse 102; temperature, 100 $\frac{4}{5}$; at 2 p. m., pulse, 124; temperature, 102. Ordered alcohol bath (sponging) and quinine, gr. x at 5 p. m., pulse 113; temperature, $101\frac{3}{5}$.

Second day, a. m., pulse 118; temperature $101\frac{4}{5}$; 10 p. m., pulse 103; temperature $100\frac{1}{5}$. Third day, a. m., pulse 113; temperature $102\frac{1}{2}$; 2:15 p. m., pulse 116; temperature $103\frac{1}{2}$. Given antifebrin gr. viii, and alcohol bath. At 7 p. m., pulse 98; temperature 101. At 11 p. m., pulse 110, small and compressible; temperature 102—although gr. x antipyrin had been given.

Fourth day, a. m., pulse 114; temperature $101\frac{6}{10}$. Pulse "rather small," antifebrin gr. x reduced temperature by a small fraction, but at 6 p. m., pulse 120; temperature 103. Liquid nourishment and whiskey given in sufficient quantity. Below the incision, and deep, a feeling of puffiness and fluctuation. Dressings not loosed, and " expectant treatment " followed.

Fifth day, a. m., pulse 114; temperature $100\frac{6}{10}$, and there appears tendency to delirium. At 7 p. m., pulse 118; temperature $102\frac{8}{10}$. Urine of morning deep red, with heavy deposit of sediment, spec. grav. 1030; albumen and sugar absent. Reaction alkaline; at 10 p. m., pulse 118; temperature 103; mind clear. Sixth day, a. m., pulse 102; temperature $99\frac{6}{10}$. To prevent rise in temperature, antifebrin gr. ix given, followed with j gr. antifebrin every hour. Fluctuation continues. P. m., edges of incision separated near center and 4 to 5 ounces bloody sero-pus escaped. Cavity drenched with carb. water, 1-40 and frequent doses quinine gr. x given; at 11 p. m., pulse 102; temperature 101. Seventh day, a .m., pulse 90; temperature $100\frac{3}{10}$. Three or four ounces pus escaped when dressings were removed. P. m. pulse 96; temperature $102\frac{4}{10}$. Eighth day, a. m., pulse 114; temperature $100\frac{6}{10}$. ℥iv sanious pus discharged; p. m., pulse 120; temperature $102\frac{6}{10}$. Ninth day, a. m., pulse 109; temperature $99\frac{4}{5}$; p. m., pulse 112; temperature $100\frac{2}{10}$. Incision made at lower part of cavity, 3 inches below sutures. Tenth day, a. m., pulse 104; temperature $99\frac{7}{10}$. Antifebrin continued; p. m., pulse 121; temperature $100\frac{8}{10}$. Considerable pus, but "healthier looking."

The patient gradually improved from this time, though continuing to have exacerbations of temperature seeming to indicate malarial complications. Pus gradually became creamy and healthy-looking—though on the fifteenth day two or three semi-decomposed fasciculi of the *pect. maj.* were "thrown off." Horse hair sutures lost their utility and edges of wound gradually attained an inch between them; having large surface which is now slowly granulating under ℞ Bals. Peruv. ʒii, Ungt. Petrolat. ℥i.

Horse hair sutures are considered non-irritating and superior to silk, by Dr. Lewis, of the cancer service. Antifebrin acted in this case better than antipyrin, and as it is cheaper, would seem a good substitute.

As "Alexander's operation," shortening of the round ligaments, is not very frequently done, I will describe it as seen recently:

The woman had complete prolapsus—the uterus protruding over two inches. Patient etherized, and incision made parallel to Pouparts ligament, three inches long, down to ring. Ligament found very small and attenuated, as it was also on right side. The uterus was now in pelvis and was drawn downward by means of tenaculum, to aid in identifying ligaments by the tension. The ligaments were cleared of investing tissue ; the uterus pushed well up; and about six inches removed from each. Four or five sutures were taken through edges of the ring, and about three of the five of each side were passed through the ligament, holding it firmly. Sutures were of cat-gut "kept" in oil of juniper. The uterus could now be distinctly felt above pubes. Edges of wounds co-aptated with deep sutures of cat-gut. On the left side, a bone drainage tube was introduced, while incision of the right side was completely closed. Iodoform collodion painted over each and iodoform gauze applied and bandaged on, after introduction of a pessary, as additional support. Within three days skin and subcutaneous tissues became œdematous ; abdomen a little swollen and tympanitic, while the bone drainage "failed to work." No great increase in pulse and temperature occurred, tympanites continued to be after bowels were moved well. Each wound now allowed drainage and the temperature, six days after operation, is only $100\frac{2}{5}°$. A very dark purulent fluid is discharged. Dressing consists in removing all pus and washing with carbolized water, subnitrate bismuth, then used to cover wound, and over this the bichloride gauze and absorbent cotton.

I hope yet to be able to report a success in the use of iodoform collodion as a "hermetical" dressing, though for some reason, the experiment failed in above cases.

### DERMATOLOGY.

During the year ending May 1, 1,400 cases were recorded as applying for treatment in the Skin and Cancer Hospital. Of these, 1,108 were seen in the Skin service, and I have made the following table as to relative frequency of the principal diseases, viz: eczema, of all types, 357; acne, 125; simple ulcer 68; syphilis, 65; psoriasis, 30; urticaria, 28; scabies, 24; rosacea, 23; pendiculi capitis, 17; ped. corp., 12; trichophytosis, cap. et corp., 10; lichen planus, 9; (lichen rub. acuminat., 0); lupus erythematosus, 9; lupus vulg., 8; sycosis parasitaria, 8; sychosis non parasit., 7; chromophytosis, 4; impetigo contagiosa, 12; impetigo simplex, 5; varicella, 3; in all equalling 800 cases. The remaining 308 are made up of cases differently designated, minor cases and those belonging to other service and referred to as not being strictly dermatological.

In the Cancer department there were recorded 292 cases. Of these, 50 were epithilioma, and the remainder were other forms of malignant disease, together with a number unsuited for this service, many of which, however, were treated, and few sent away. Most of the books give the age, sex and occupation, most predisposing to the diseases mentioned when they can, but exact statistics as to *frequency are not so easy to obtain.*

*Phlebarterioectasia.*—This is the diagnosis of the following described case:

Woman, aged 32. Condition of varix back of left hand since childhood, increasing in past five years. At present ulnar side of dorsum of hand ⅔ whole dorsum the seat of raised, fluctuating, cavernous tumor, filled with blood and yielding upon pressure. First phalanx index finger, second of ring and at juncture of first and second of little finger, a similar condition, nearly surrounding fingers. Phlebolith over metacarpal bone of little finger size of small hazlenut. Treatment belongs to some surgeon, case being referred—*after above diagnosis.*

M. B. HUTCHINS, M. D.

